# A nasal vaccine with inactivated whole-virion elicits protective mucosal immunity against SARS-CoV-2 in mice

**DOI:** 10.3389/fimmu.2023.1224634

**Published:** 2023-08-31

**Authors:** Nagisa Tokunoh, Shigeyuki Tamiya, Masato Watanabe, Toru Okamoto, Jessica Anindita, Hiroki Tanaka, Chikako Ono, Toshiro Hirai, Hidetaka Akita, Yoshiharu Matsuura, Yasuo Yoshioka

**Affiliations:** ^1^ Innovative Vaccine Research and Development Center, The Research Foundation for Microbial Diseases of Osaka University, Osaka, Japan; ^2^ Vaccine Creation Group, BIKEN Innovative Vaccine Research Alliance Laboratories, Research Institute for Microbial Diseases, Osaka University, Suita, Osaka, Japan; ^3^ Department of Microbiology and Immunology, School of Pharmaceutical Sciences, Wakayama Medical University, Wakayama, Wakayama, Japan; ^4^ Institute for Advanced Co-Creation Studies, Research Institute for Microbial Diseases, Osaka University, Osaka, Japan; ^5^ Center for Infectious Disease Education and Research, Osaka University, Suita, Osaka, Japan; ^6^ Laboratory of DDS Design and Drug Disposition, Graduate School of Pharmaceutical Sciences, Tohoku University, Sendai, Miyagi, Japan; ^7^ Laboratory of DDS Design and Drug Disposition, Graduate School of Pharmaceutical Science, Chiba University, Chiba-shi, Chiba, Japan; ^8^ Laboratory of Virus Control, Research Institute for Microbial Diseases, Osaka University, Suita, Osaka, Japan; ^9^ BIKEN Innovative Vaccine Research Alliance Laboratories, Institute for Open and Transdisciplinary Research Initiatives, Osaka University, Suita, Osaka, Japan; ^10^ Laboratory of Nano-design for Innovative Drug Development, Graduate School of Pharmaceutical Sciences, Osaka University, Suita, Osaka, Japan; ^11^ Center for Advanced Modalities and DDS, Osaka University, Suita, Osaka, Japan; ^12^ Global Center for Medical Engineering and Informatics, Osaka University, Suita, Osaka, Japan

**Keywords:** antigen, IgA, inactivated whole-virion, messenger RNA vaccine, nasal vaccine, SARS-CoV-2, upper respiratory tract

## Abstract

**Introduction:**

Vaccinations are ideal for reducing the severity of clinical manifestations and secondary complications of severe acute respiratory syndrome coronavirus 2 (SARS-CoV-2); however, SARS-CoV-2 continues to cause morbidity and mortality worldwide. In contrast to parenteral vaccines such as messenger RNA vaccines, nasal vaccines are expected to be more effective in preventing viral infections in the upper respiratory tract, the primary locus for viral infection and transmission. In this study, we examined the prospects of an inactivated whole-virion (WV) vaccine administered intranasally against SARS-CoV-2.

**Methods:**

Mice were immunized subcutaneously (subcutaneous vaccine) or intranasally (nasal vaccine) with the inactivated WV of SARS-CoV-2 as the antigen.

**Results:**

The spike protein (S)-specific IgA level was found to be higher upon nasal vaccination than after subcutaneous vaccination. The level of S-specific IgG in the serum was also increased by the nasal vaccine, although it was lower than that induced by the subcutaneous vaccine. The nasal vaccine exhibited a stronger defense against viral invasion in the upper respiratory tract than the subcutaneous vaccine and unimmunized control; however, both subcutaneous and nasal vaccines provided protection in the lower respiratory tract. Furthermore, we found that intranasally administered inactivated WV elicited robust production of S-specific IgA in the nasal mucosa and IgG in the blood of mice previously vaccinated with messenger RNA encoding the S protein.

**Discussion:**

Overall, these results suggest that a nasal vaccine containing inactivated WV can be a highly effective means of protection against SARS-CoV-2 infection.

## Introduction

Severe acute respiratory syndrome coronavirus 2 (SARS-CoV-2) has caused a pandemic with over > 760 million cases and > 6.9 million deaths worldwide as of April 2023, according to World Health Organization reports. SARS-CoV-2 infection leads to a clinical syndrome ranging from mild to severe Coronavirus Disease 2019 (COVID-19), including acute lung injury and acute respiratory disease syndrome (1). SARS-CoV-2 infection is also reported to cause strokes, myocarditis, heart failure, and hyperinflammatory shock syndrome (2–4). High-risk groups, including the elderly and those with chronic respiratory disease, are at an increased risk of severe COVID-19, which is associated with respiratory failure and death (1, 5).

Vaccination is a key strategy to prevent pneumonia, secondary complications, and mortality from SARS-CoV-2 infection at the population level, especially among the elderly and those with chronic respiratory disease (6, 7). To date, injectable vaccines such as messenger RNA (mRNA) vaccines, adenovirus vector-based vaccines, subunit vaccines, and inactivated whole-virion (WV) vaccines against SARS-CoV-2 have been developed and used worldwide (8–11). In particular, mRNA vaccines administered intramuscularly to people worldwide have been effective in reducing disease severity and symptomatic cases (12–14).

Human SARS-CoV-2 infects the upper respiratory tract causing mild disease; however, its progression to the lower respiratory tract leads to pneumonia and serious disease (15, 16). Further, the upper respiratory tract is an important site for the initiation of viral transmission through coughing, sneezing, and talking (15, 16). In the early phase of SARS-CoV-2 infection, the spike (S) protein specifically binds to cellular entry receptors such as angiotensin-converting enzyme 2, which is less expressed in the lungs compared with that in the upper respiratory tract in humans (17–19). Previous reports have shown that during a human challenge with SARS-CoV-2, the virus replicates at significantly higher levels in the nose (20); however, most of the current SARS-CoV-2 vaccines which are administered parenterally *via* intramuscular injection, fail to induce virus-specific IgA production in the upper respiratory tract, even though they robustly induce production of virus-specific IgG in the blood (21–23). In fact, these vaccines can protect against COVID-19 while providing limited protection against respiratory virus replication and shedding in the upper respiratory tract in non-human primates (24–26). Collectively, these parenteral vaccines are not fully effective in preventing SARS-CoV-2 infection of the upper respiratory tract, though virus-specific IgG in blood may protect against disease exacerbation. Therefore, blocking the initial infection, generation, and shedding of SARS-CoV-2 in the upper respiratory tract is critical for vaccine development.

In contrast to parenteral vaccines, nasally administered vaccines (nasal vaccines) induce the production of antigen-specific IgG and IgA in the blood and respiratory tract, respectively (27, 28). To date, virus-specific IgA in the upper respiratory tract induced by nasal vaccines has shown protection against influenza virus and SARS-CoV-2 infection more efficiently than that with parenteral vaccines (29–34). For example, Hemmi et al. showed that virus-specific IgA induced by a nasal vaccine containing the recombinant S protein from SARS-CoV-2 adjuvanted with CpG oligonucleotides was highly protective against SARS-CoV-2 challenge in the upper respiratory tract of mice (33). In addition, nasal vaccine using the recombinant receptor binding domain of S protein from SARS-CoV-2 adjuvanted with Riboxxim, a Toll-like receptor (TLR)3 ligand, induced robust mucosal IgA antibody in murine models (34). Furthermore, virus-specific T cell responses, particularly respiratory tissue-resident memory CD8^+^ T cells and CD4^+^ T cells, induced by nasal vaccine and infection protect against severe disease and mortality induced by influenza virus and SARS-CoV-2 (35–37), although T cell responses induced by the parenteral vaccine are limited to peripheral locations, failing to either access or be maintained at the respiratory mucosa (35). Therefore, a nasal vaccine could be a highly effective means of preventing SARS-CoV-2 infection in the respiratory tract.

Nasal vaccines based on adenovirus vectors and subunit vaccines against SARS-CoV-2 have been developed in preclinical studies and clinical trials (38). Recent research indicates that the intranasal administration of AstraZeneca’s ChAdOx-1 vaccine has poor immunogenicity in humans previously immunized with mRNA vaccines, despite being effective in mice (35, 39). In addition, these vaccines have several limitations. For example, adenovirus vector-based vaccines are ineffective at inducing adenovirus-specific antibodies following the first dose (40). Subunit vaccines require adjuvants to elicit immune responses (41, 42) because intranasal administration of antigens alone does not elicit antigen-specific antibodies. However, no approved adjuvants for nasal vaccines in humans exist, although several adjuvants, such as c-di-GMP, CpG oligonucleotides, poly(I:C), and Riboxxim, have been experimentally implemented in nasal vaccines (34, 43–45).

Inactivated WVs have shown high preventive efficacy, preservation, and transport, and retain sufficient antigenic properties to elicit immune responses (46, 47). Previous reports on influenza viruses have shown that inactivated WV induces strong immune responses due to strong adjuvant activity because inactivated WV contains single-stranded viral RNA (ssRNA), which acts as a ligand for TLR7 (48, 49). In SARS-CoV-2, ssRNA also acts as an activator of TLR7/8 to activate inflammation and immunity (50), indicating strong adjuvant activity of inactivated WV of SARS-CoV-2. To date, parenteral inactivated WVs are available against SARS-CoV-2, and have shown promise in preclinical and clinical studies, leading to approval for their use (51, 52). However, nasal administration of these vaccines remains to be explored. Therefore, in this study, we focused on using the inactivated WV as a nasal vaccine against SARS-CoV-2. We examined the potential of an inactivated WV as an antigen to induce S-specific IgA in the nose and S-specific IgG in blood, as well as to prevent viral infection in both the upper and lower respiratory tracts. We also examined the potential of the nasal inactivated WV as a booster mice previously vaccinated with an mRNA vaccine expressing the S protein.

## Materials and methods　

### Mice

Specific pathogen-free 10 week-old BALB/c mice were purchased from SLC (Hamamatsu, Japan). The mice were housed in a room with a 12-h:12-h light:dark cycle (lights on, 8:00 am; lights off, 8:00 pm) and unrestricted access to food and water. All animal experiments with or without SARS-CoV-2 were conducted in accordance with the guidelines of Osaka University for the ethical treatment of animals and were approved by the Animal Care and Use Committee of the Research Institute for Microbial Diseases, Osaka University, Japan (approval numbers: BIKEN-AP-R02-09-0).

### Virus

The Gamma strain (hCoV-19/Japan/TY7-503/2021) of SARS-CoV-2 was obtained from the National Institute of Infectious Diseases (Tokyo, Japan). The virus was expanded in VeroE6/TMPRSS2 cells (JCRB Cell Bank, Osaka, Japan) and stored at −80 °C until use. VeroE6/TMPRSS2 cells were cultured in DMEM (Nacalai Tesque, Kyoto, Japan) containing 10% fetal calf serum (FCS) and 1% penicillin/streptomycin. Mice were intranasally challenged with MA10, a mouse-adapted SARS-CoV-2. MA10 was generated using a circular polymerase extension reaction as described previously (53). The SARS-CoV-2 NIID strain (2019-nCoV_Japan_TY_WK-5212020) served as the backbone of MA10. MA10 contains seven mutations, which were introduced adaptively into SARS-CoV-2 during serial passages in BALB/c mice (54). Each experiment with live SARS-CoV-2 was performed within a biosafety level 3 facility at Osaka University, adhering to stringent guidelines.

### Preparation of the inactivated whole-virion vaccine

VeroE6/TMPRSS2 cells in 225 cm^2^ flasks (Corning, Corning, NY, USA) were infected with the Gamma strain of SARS-CoV-2 at 0.01 multiplicity of infection (MOI) at 37 °C. After three days, the cell culture medium was collected *via* centrifugation at 3000 rpm for 10 min to remove debris. Live SARS-CoV-2 in the supernatant was inactivated using β-propiolactone (Wako, Osaka, Japan) at a concentration of 1:4000 (v/v) at 4 °C for 24 h, then incubated at 37 °C for 2 h to hydrolyze the remaining β-propiolactone. To confirm viral inactivation, we performed a plaque assay before purification. Briefly, VeroE6/TMPRSS2 cell monolayers (1 × 10^6^ cells/well) in 6-well plates (Corning) were incubated with medium as a negative control, live SARS-CoV-2 (diluted 10^4^-fold with medium) as a positive control, or inactivated WV (undiluted) at 37 °C for 2 h. The cells were then rinsed twice with phosphate-buffered saline (PBS) and covered with agar (final 1%; SeaPlaque TM Agarose, Lonza, Basel, Switzerland) in a medium containing 2% FCS and 0.5% penicillin/streptomycin. The plates were then incubated at 37 °C. After three days, the cells were fixed with 10% formalin (Nacalai Tesque) and the agar was removed. The cells were then stained with 1% crystal violet (Tokyo Chemical Industry, Tokyo, Japan) and the plaques were counted. Next, the inactivated WV was purified using sucrose density gradient centrifugation. Briefly, using 20% sucrose, inactivated WV pellets were collected by centrifugation at 32,000 rpm at 4 °C for 2 h. The pellets were resuspended in PBS, and to exclude artefacts, purified through a 15–60% sucrose gradient at 30,000 rpm at 4 °C for 2 h. After centrifugation, 23 fractions (1.5 mL each) were collected from the top to bottom, and the expression of S protein (16–19 fractions) was confirmed by western blotting. Subsequently, to exclude sucrose, 16-19 fractions were diluted with PBS and centrifuged at 30,000 rpm at 4 °C for 3 h. Finally, the pellets were resuspended in PBS and the amount of inactivated WV was quantified using a Pierce protein assay kit (Thermo Fisher Scientific, Hampton, NH, USA) with a bovine serum albumin standard.

### Confirmation of viral inactivation *in vivo*

To confirm viral inactivation *in vivo*, mice were treated with SARS-CoV-2 (Gamma strain; 1.7 × 10^5^ Plaque-forming unit; PFU), SARS-CoV-2 MA10 (2 × 10^5^ PFU), or inactivated WV (3 μg or 10 μg) in 20 μL of PBS under anesthesia. The nasal turbinate was harvested and homogenized in DMEM containing 2% FCS and 1% penicillin/streptomycin 3 days after treatment, followed by centrifugation and supernatant collection. To titrate the infectious virus, the supernatant of the nasal turbinate was serially diluted in DMEM containing 2% FCS and 1% penicillin/streptomycin. The plaque assay was performed as described above.

### SDS-PAGE and western blotting

Sodium dodecyl sulfate-polyacrylamide gel electrophoresis (SDS-PAGE) was performed in accordance with previous publications (32). Briefly, purified proteins were mixed in sample buffer solution (Nacalai Tesque) containing 2-mercaptoethanol (Sigma-Aldrich, St. Louis, MO, USA) at 1:1 (v/v) ratio and heated at 95 °C for 5 min before being loaded onto a 10% Mini-PROTEAN TGX Precast Protein Gel (Bio-Rad, Hercules, CA, USA). Following electrophoresis, the gels were stained with Coomassie Brilliant Blue (Nacalai Tesque). After gel electrophoresis, protein bands were transferred from the SDS-PAGE gel onto polyvinylidene fluoride membranes (Bio-Rad). After blocking in 5% skim milk (w/v) diluted in distilled water, the transferred membrane was washed thrice with PBST (0.05% Tween-20 in PBS) and incubated with mouse anti-SARS-CoV-2 (2019-nCoV) spike antibody (clone: #42; Sino Biological, Beijing, China) or rabbit anti-SARS membrane protein antibody (polyclonal; Novus Biologicals, Littleton, CO, USA). The membrane was then washed thrice with PBST, and incubated with a rabbit anti-mouse horseradish peroxidase-conjugated secondary antibody (Abcam Ltd., Cambridge, UK) or goat anti-rabbit horseradish peroxidase-conjugated secondary antibody (Abcam Ltd.), and detected using a ChemiDoc Touch Imaging System (Bio-Rad). The amount of S protein in inactivated WV was determined using western blotting (**Supplementary Figure 1B**). We used recombinant S protein as standard (from 4.3 ng/well to 69.3 ng/well). After SDS-PAGE, the proteins were transferred onto the membrane for western blotting. The transferred membrane was incubated with rabbit anti-SARS-CoV-2 (2019-nCoV) spike antibody (Polyclonal IgG; Sino Biological). The membrane was then washed thrice with PBST and incubated with a goat anti-rabbit horseradish peroxidase-conjugated secondary antibody (Abcam Ltd.). The three points (34 ng/well, 68 ng/well, and 136 ng/well) for inactivated WV were averaged. Immunoreactive bands were quantified using Image J software (NIH Image, Bethesda, MD, USA).

### Negative stain electron microscopy

The inactivated WV was directly analyzed using a Tecnai G2 20 twin transmission electron microscopy (TEM; FEI Company, Hillsboro, OR, USA) at 200 kV. Carbon-coated copper grids with 250 mesh (STEM Co. Ltd, Tokyo, Japan) were glow-discharged using DII-29020HD (JEOL, Tokyo, Japan). The inactivated WV was fixed in 2% glutaraldehyde overnight at 4 °C. Samples were diluted in PBS to 20 μg/mL immediately before measurement, and 10 μL of the sample was applied to the grids for 5 min. Four-diluted EM stainer (Nisshin EM, Tokyo, Japan) was then used to stain the sample on the grid for 30 min. The specimen was finally gently blotted from the side with filter paper and air-dried before imaging. Images were recorded on an Eagle 2 K × 2 K CCD camera with a defocus of -2 μm at a nominal magnification of 25,000× or 50,000×.

### Expression and purification of the recombinant S protein

The cDNA of the S protein ectodomain from the Gamma strain of SARS-CoV-2 with a C-terminal hexahistidine tag (His-tag) was cloned into the pcDNA3.1 expression plasmid (Thermo Fisher Scientific). The foldon sequence (GYIPEAPRDGQAYVRKDGEWVLLSTFL) derived from the fibritin of bacteriophage T4 was integrated at the C terminus of the S protein. S protein production was performed in accordance with previous publications (32).

### Preparation of mRNA vaccine

For the transcription of mRNA expressing the S protein of SARS-CoV-2, template pDNA encoding the S protein (Wuhan-Hu-1) was prepared using polymerase chain reaction. The pDNA-spike was linearized using restriction enzymes. After phenol-chloroform extraction and ethanol precipitation, the linearized pDNA was transcribed into mRNA using the MEGAscript™ T7 transcription kit (Thermo Fisher Scientific) according to the manufacturer’s instructions. N1-methylpseudouridine was incorporated into the mRNA by replacing uridine. Residual dsRNA was removed as previously described (55). The 5′ cap was added according to the ScriptCap Cap 1 Capping System protocol (Madison, WI, USA). The 3′ poly(A) tail was added according to the protocol provided with the poly(A) Tailing Kit (Thermo Fisher Scientific). A lipid mixture containing SM-102 (Cayman Chemical Company, Ann Arbor, MI, USA), DMG-PEG2000 (NOF Corporation, Tokyo, Japan), DSPC (NOF Corporation), and cholesterol (Sigma-Aldrich) in an ethanol solution (SM-102/DSPC/cholesterol/DMG-PEG2000 = 50/10/38.5/1.5) were prepared. The mRNA-lipid nanoparticles (LNPs) were produced by mixing the mRNA dissolved in acetic acid/NaOH buffer (pH 5.0) and the lipid mixture in ethanol at an N/P ratio of 5.5 using a NanoAssemblr^®^ instrument (Precision Nanosystems, Vancouver, Canada) at a flow rate ratio of 3:1. The external solution of the mRNA-LNPs was replaced with PBS (Nacalai Tesque) by ultrafiltration using Amicon Ultra-4-100K centrifugal units. The size and zeta potential of mRNA-LNPs were measured using a Zetasizer Nano ZS (Malvern Instruments, Malvern, UK). The encapsulation efficiency of mRNA was measured using the RibogreenTM reagent (Invitrogen, Carlsbad, CA, USA).

### Vaccination

For nasal vaccination, BALB/c mice were administered with inactivated WV (3 μg/mouse) in a total volume of 5 μL, divided equally between both nostrils. Subcutaneous vaccination was achieved *via* administration of the BALB/c mice at the base of the tail with inactivated WV (3 μg/mouse) combined with Alhydrogel (alum; InvivoGen, San Diego, CA, USA) as an adjuvant (50 μg/mouse) in a total volume of 50 μL. For both routes, the mice were administered on days 0 and 21. Unimmunized mice were used as controls. On day 32, serum, nasal wash, and bronchoalveolar lavage fluid (BALF) samples were collected and stored at -80 °C. The nasal cavity was gently flushed using 400 μL of PBS to obtain nasal wash samples. BALF was obtained *via* lung lavage with 1.2 mL PBS. In some experiments, mRNA expressing the SARS-CoV-2 Spike (1 μg/mouse) was administered intramuscularly to mice in a total volume of 50 μL. On day 21 after mRNA vaccination, the mice were intranasally administered with inactivated WV (3 μg/mouse) in a total volume of 5 μL, divided equally between both nostrils. On day 28, serum and nasal wash samples were collected.

### Detection of antigen-specific antibodies

We determined the levels of S-specific antibodies in the serum, nasal wash, and BALF using enzyme-linked immunosorbent assay (ELISA). To detect SARS-CoV-2-specific total IgG and IgA, ELISA plates (Corning, Corning, NY, USA) were coated overnight at 4°C with S protein of the Gamma strain in PBS (1 μg/mL or 10 μg/mL). ELISA was performed in accordance with previous publications (32). Endpoint titers were determined using the following procedure: The background value was subtracted from the OD value. A subtracted OD value of 0.1 or more was regarded as positive, and the maximum dilution to give a positive result was used as the endpoint titer.

### Analysis of neutralizing antibody titer

For the neutralization assay, VeroE6/TMPRSS2 cells (1.2 × 10^4^ cells/well) were added to a 96-well half-white plate (Greiner BIO-ONE, Kremsmunster, Austria) and incubated in DMEM containing 10% FCS and 1% penicillin/streptomycin at 37°C for 24 h. On the day after incubation, serum samples and nasal wash samples were evaluated using 50-819,200-fold, or 4-65,536-fold serial dilutions of undiluted solutions. The serial dilutions were mixed 1:1 with pseudotyped viruses, replication-deficient vesicular stomatitis virus (VSV) bearing Gamma spike of SARS-CoV-2, and incubated at 37°C for 1 h. After removing the growth medium from the cells, 50 μL of serum/virus or nasal wash/virus mixture was added to the cells and then incubated at 37°C for 48 h. Following 48 h post-infection, 50 μL of ONE-Glo™ EX Reagent (Promega, Madison, WI, USA) was pipetted into each well and mixed. Luminescence was measured using a microplate reader (CORONA electric, SH-9000, Hitachi High-Tech Corporation, Tokyo, Japan).

### Flow cytometry

To evaluate the percentage of germinal center (GC) B cells in the nasal passage, lymphocytes were collected seven days after the last immunization and analyzed *via* flow cytometry. The nasal passage lymphocytes were added to a 96-well plate, and incubated with an anti-mouse CD16/CD32 antibody (1:400 dilution; clone:93; BioLegend, San Diego, CA, USA), PE anti-mouse CD45 antibody (1:200 dilution; clone:30-F11; BioLegend), BV421 anti-mouse B220 antibody (1:200 dilution; clone: RA3-6B2; BioLegend), AF647 anti-mouse GL7 antibody (1:200 dilution; clone: GL7; BioLegend), and PE/Cy7 anti-mouse CD95 (Fas) antibody (1:200 dilution; clone: SA367H8; BioLegend) in PBS with 2% FCS, 1 mM EDTA (DOJINDO, Kumamoto, Japan), and 0.05% azide (Wako) for 30 min at 4°C. GC B cells were defined as CD45+ B220+ Fas+ GL7+ cells. Flow cytometry was performed using the CytoFLEX Flow Cytometer (Beckman Coulter, Brea, CA, USA). Kaluza software (Beckman Coulter) was used for analyzing the flow cytometry data.

### Cytokine production from splenocytes after vaccination

On day 32 after vaccination, splenocytes (1 × 10^6^ cells/well) were added to a 96-well plate. Subsequently, the cells were stimulated for three days at 37 °C with S protein or left unstimulated (final concentration: 20 μg/mL). Post-incubation, the levels of interferon (IFN)-γ and interleukin (IL)-13 in the supernatants were analyzed using commercial ELISA kits (BioLegend for IFN-γ; R&D Systems, Minneapolis, MN, USA, for IL-13), as per manufacturer’s instructions. Standards supplied with the kits were used to quantify the cytokine levels.

### Upper respiratory tract infection

Fourteen days after the last immunization, the mice were challenged intranasally with SARS-CoV-2 MA10 (5 × 10^4^ PFU) in 5 μL of PBS under anesthesia. Unimmunized mice were used as controls. After challenge, the nasal turbinates were harvested and homogenized in 500 μL of DMEM containing 2% FCS and 1% penicillin/streptomycin. Next, 500 μL of DMEM containing 2% FCS and 1% penicillin/streptomycin was added, followed by centrifugation and collection of the supernatant. To titrate the infectious virus, the supernatant of the nasal turbinate was serially diluted in DMEM containing 2% FCS and 1% penicillin/streptomycin. The plaque assay was performed as previously mentioned.

### Lower respiratory tract infection

Fourteen days after the last immunization, mice were infected intranasally with SARS-CoV-2 MA10 (5 × 10^4^ PFU or 2 × 10^5^ PFU) in 20 μL of PBS under anesthesia. Unimmunized mice were used as controls. After the infection, the body weight and survival of the mice were observed for eight days. The day of death was defined as the specific day on which the body weight of the mice fell below 75% of their initial weight.

### Statistical analyses

Statistical analyses were performed using Prism software (GraphPad Software, San Diego, CA, USA). All data are presented as mean ± standard deviation (SD). One-way ANOVA was used to determine significance differences, followed by Tukey’s test. *P* < 0.05 was considered to indicate statistical significance.

## Results

### Preparation of inactivated whole-virion vaccine for SARS-CoV-2

**Figure 1:**
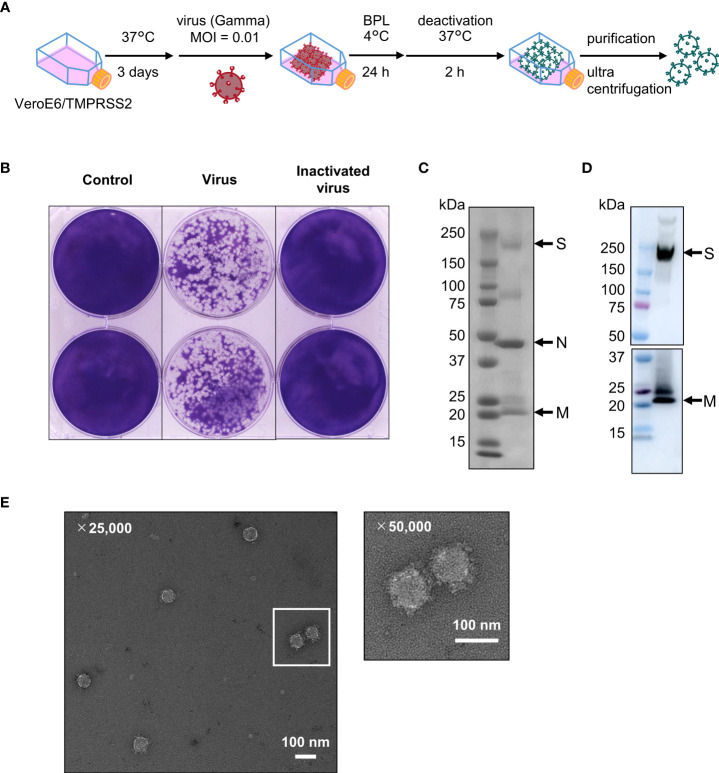
Inactivated whole-virion (WV) vaccine retains the protein composition and the structure of the original virus. **(A)** Scheme of inactivated WV (Gamma strain) production. **(B)** VeroE6/TMPRSS2 cells were treated with medium as a negative control, inactivated WV (undiluted) and live virus (diluted 10^4^-fold with medium) at 37 °C for 3 days, followed by plaque counting. **(C, D)** Inactivated WV were validated using **(C)** SDS-PAGE; left: marker, right: sample and **(D)** western blotting; left: marker, right: S protein and M protein. Each experiment was performed in duplicate. **(E)** TEM micrograph of SARS-CoV-2 virus particles, Scale bar, 100 nm.

To generate inactivated WV, we used a Gamma strain (hCoV-19/Japan/TY7-503/2021) as the seed virus. The virus was propagated in VeroE6 cells expressing transmembrane serine protease 2 (TMPRSS2) and was inactivated using β-propiolactone (**Figure 1A**). Using a plaque formation assay, we confirmed that β-propiolactone completely inactivated SARS-CoV-2 (**Figure 1B**). In addition, no live virus was detected in the nasal turbinates of mice intranasally administrated inactivated WV using plaque formation assay (**Supplementary Figure 1A**). Inactivated WV was purified using sucrose density gradient centrifugation and the purity of the inactivated WV was confirmed using SDS-PAGE (**Figure 1C**) and western blotting (**Figure 1D**). SDS-PAGE analysis revealed three major bands. Bands of approximately 150, 50, and 15 kDa indicated S protein, nucleocapsid (N) protein, and membrane (M) protein, respectively (56). Western blot analysis also showed bands for the S and M proteins (**Figure 1D**). Next, we evaluated the amount of S protein in inactivated WV using western blotting. As a result, we found that 3 μg of inactivated WV contained approximately 2.1 μg of S protein (**Supplementary Figure 1B**). Furthermore, using transmission electron microscopy (TEM; **Figure 1E**), we demonstrated that the inactivated WV retained the structure of the virus. These results suggest that inactivated WV could be used as a vaccine antigen.

### Antibody responses induced by the nasal inactivated whole-virion vaccine

**Figure 2:**
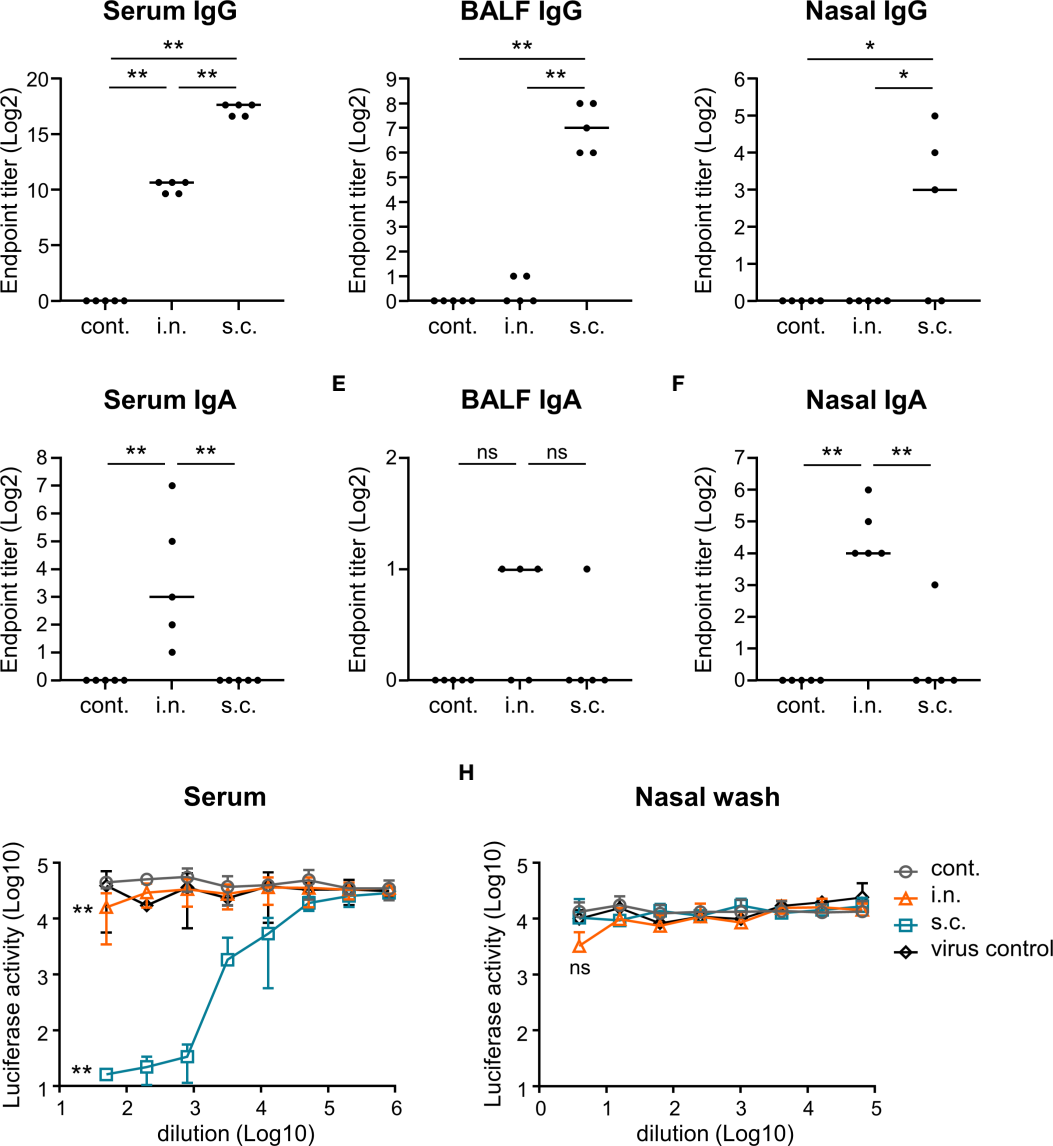
Nasal administration of the inactivated whole-virion (WV) vaccine induces S protein-specific IgG in serum and IgA in nasal wash. **(A–H)** Mice were immunized twice with inactivated WV intranasally (i.n.) or with inactivated WV and alum subcutaneously (s.c.). Unimmunized mice were used as controls (cont.). **(A–F)** Endpoint titers of S protein-specific IgG in **(A)** serum, **(B)** bronchoalveolar lavage fluid (BALF), and **(C)** nasal wash, and endpoint titers of S protein-specific IgA in **(D)** serum, **(E)** BALF, and **(F)** nasal wash were evaluated. **(G, H)** Measurement of neutralization against vesicular stomatitis virus-based pseudotyped viruses displaying Gamma spike of SARS-CoV-2 in **(G)** serum samples and **(H)** nasal wash samples. The significance of differences in the **(G)** 50-fold-diluted serum samples and **(H)** 4-fold-diluted nasal wash samples was evaluated. The data is presented as mean ± SD. Each experiment was performed in duplicate. **(A–H)** n = 5 per group. **(A–H)** *P < 0.05; **P < 0.01 as indicated by Tukey’s test. ns, not statistically significant.

**Figure 3:**
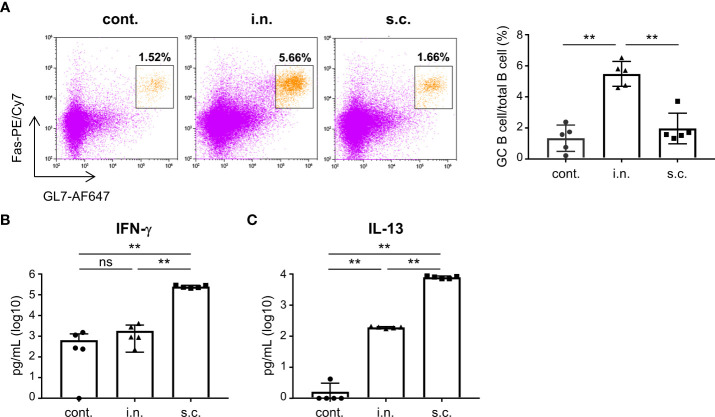
Nasal administration of the inactivated whole-virion (WV) vaccine induces germinal center (GC) B cells in the nasal passage. **(A–C)** Mice were immunized twice with inactivated whole-virion (WV) vaccine intranasally (i.n.) or with inactivated WV and alum subcutaneously (s.c.). Unimmunized mice were used as controls (cont.). **(A)** The percentage of germinal center (GC) B cells (CD45+ B220+ Fas+ GL7+) among the total B cells in the nasal passage were measured using flow cytometry. **(B, C)** Splenocytes were cultured in the presence or absence of S protein for three days, after which the levels of **(B)** IFN-γ and **(C)** IL-13 in the supernatant were measured using ELISA. The data is presented as mean ± SD. Each experiment was performed in duplicate. **(A–C)** n = 5 per group. **(A–C)** **P < 0.01 as indicated by Tukey’s test.

To evaluate the functionality of nasal vaccines, mice were immunized intranasally (nasal vaccine) with inactivated WV without an adjuvant or subcutaneously (subcutaneous vaccine) with inactivated WV and alum as an adjuvant. The endpoint titers of S protein-specific IgG and IgA in the serum, bronchoalveolar lavage fluid (BALF), and nasal washes after vaccination were analyzed using ELISA. The endpoint titers of S-specific IgG in the serum of the nasal vaccine group were significantly higher than those in the unimmunized control group but lower than those in the subcutaneous vaccine group (**Figure 2A**). Subcutaneous vaccines induced significantly higher levels of S-specific IgG in the serum, BALF, and nasal washes than those with nasal vaccines (**Figures 2A–C**). In contrast, the endpoint titers of S-specific IgA in the serum (**Figure 2D**) and nasal wash (**Figure 2F**) were significantly higher in the nasal vaccine group than in the subcutaneous vaccine and unimmunized control groups, although no change was observed in BALF (**Figure 2E**). To examine neutralizing antibody titer in serum and nasal wash from mice vaccinated with inactivated WV, we used a pseudotyped virus displaying a Gamma spike of SARS-CoV-2. The serum from intranasally and subcutaneously vaccinated mice neutralized the pseudotyped virus, whereas the serum from non-vaccinated mice was ineffective (**Figure 2G**). In contrast, the nasal wash from intranasally vaccinated mice tended to neutralize compared to that from subcutaneously vaccinated mice and non-vaccinated mice (**Figure 2H**). Next, to evaluate germinal center (GC) B cells in the nasal passage induced by vaccination, we examined the percentage of GC B cells among total B cells using flow cytometry (**Figure 3A**; **Supplementary Figure 2**). Seven days after the last immunization, the percentage of GC B cells among the total B cells was significantly higher in the nasal vaccine group than in the subcutaneous vaccine and unimmunized control groups (**Figure 3A**). These results indicated that the inactivated WV nasal vaccine strongly induced S-specific IgA in the upper respiratory tract.

### T cell responses induced by vaccine

To examine the SARS-CoV-2-specific CD4+ T cell response induced by vaccination, we examined cytokine production in splenocytes. Splenocytes from vaccinated mice were stimulated with S protein, and the amount of cytokines in the supernatant was analyzed. The levels of IFN-γ and IL-13 (cytokines associated with T helper type 1 and 2 cells, respectively) produced by splenocytes after S protein stimulation were significantly higher in the subcutaneous vaccination group than in the nasal vaccination and unimmunized control groups (**Figures 3B, C**). In contrast, there was no difference in the level of IFN-γ between the unimmunized control and nasal vaccine groups, whereas the level of IL-13 in the nasal vaccine group was significantly higher than that in the unimmunized controls (**Figures 3B, C**).

### Virus challenge after vaccination

**Figure 4:**
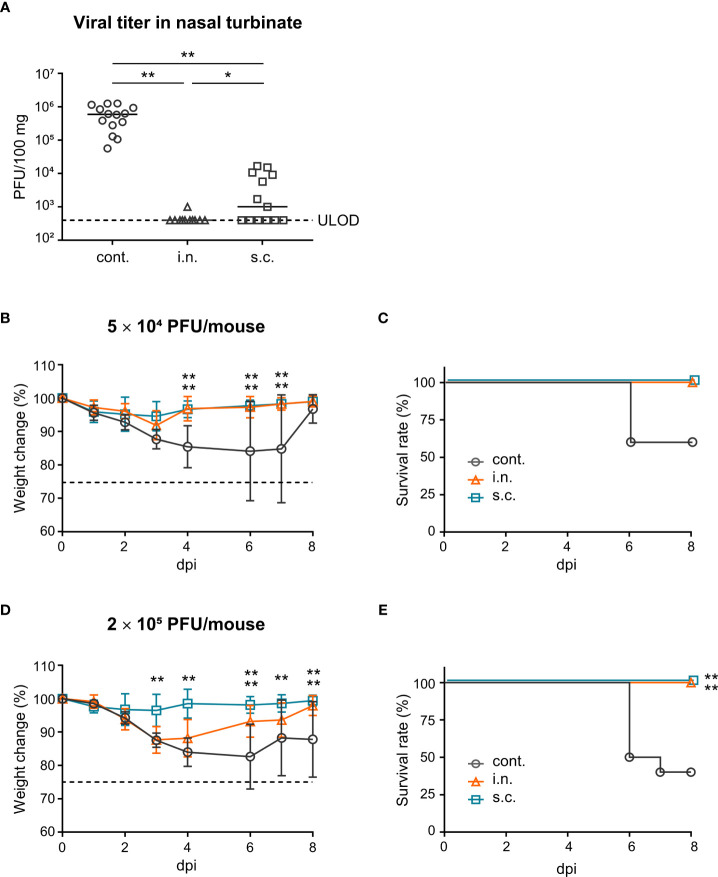
Nasal administration of the inactivated whole-virion (WV) vaccine protects against upper and lower respiratory tract infections caused by SARS-CoV-2. **(A–E)** Mice were immunized twice with inactivated whole-virion (WV) vaccine intranasally (i.n.) or with inactivated WV and alum subcutaneously (s.c.). Unimmunized mice were used as controls (cont.). **(A)** Mice were intranasally infected with SARS-CoV-2 MA10 (5 × 10^4^ PFU) in 5 μL of PBS and the SARS-CoV-2 titer in nasal turbinate was assayed using the plaque assay. **(B–E)** Mice were intranasally infected with SARS-CoV-2 MA10 (**B, C**; 5 × 10^4^ PFU/mouse, **D, E**; 2 × 10^5^ PFU/mouse) in 20 μL of PBS; **(B, D)** body weight loss and **(C, E)** survival were monitored. The data is presented as the mean ± SD. Each experiment was performed in duplicate. **(A)** n = 5 per group. **(B, C)** n = 4–5 per group. **(D, E)** n = 10 per group. **(A, B, D)** *P < 0.05; **P < 0.01 as indicated by Tukey’s test. **(C, E)** **P < 0.01 vs. the control group as indicated by comparing Kaplan–Meier curves using the Log rank test.

To evaluate the defensive effects of nasal vaccination on the upper respiratory tract, vaccinated mice were challenged intranasally with a mouse-adapted strain of SARS-CoV-2 (SARS-CoV-2 MA10; 5 × 10^4^ PFU) (54) in a total volume of 5 μL. In this model, a plaque assay was used to measure the viral titer in the nasal turbinates as an indicator of vaccination effectiveness. After challenge, the viral titer in the nasal turbinates of the nasal vaccine group was significantly lower than that in the subcutaneous vaccine and unimmunized control groups (**Figure 4A**). In addition, the virus titer in the nasal turbinates of the subcutaneous vaccine group was significantly lower than that in the unimmunized control group (**Figure 4A**). Furthermore, to assess the defensive effects of nasal vaccination on the lower respiratory tract, vaccinated mice were challenged intranasally with SARS-CoV-2 MA10 (5 × 10^4^ PFU or 2 × 10^5^ PFU) in a total volume of 20 μL. In this model, body weight loss and survival were measured as indicators of vaccination effectiveness and these parameters were monitored for eight days after infection. At a low-titer challenge (5 × 10^4^ PFU), the unimmunized controls lost weight and some of them died after the challenge, whereas mice in the nasal vaccine and subcutaneous vaccine groups did not (**Figures 4B, C**). In addition, at a high titer challenge (2 × 10^5^ PFU), the subcutaneous vaccine group completely protected from weight loss and death (**Figures 4D, E**). The nasal vaccine group was also completely protected from death, even though it showed a body weight decrease (**Figures 4D, E**). These results suggest that vaccination with intranasally inactivated WV can protect against upper and lower respiratory tract infections caused by SARS-CoV-2.

### Systemic priming by mRNA vaccine followed by intranasal boosting with inactivated WV

**Figure 5:**
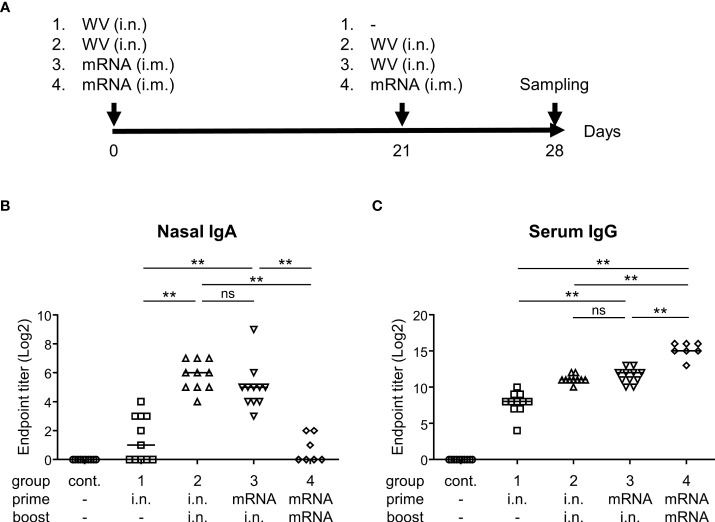
Systemic priming by mRNA vaccine followed by intranasal boosting with inactivated whole-virion (WV) vaccine induces S protein-specific IgG in serum and IgA in nasal wash. **(A–C)** Mice were immunized intranasally (i.n.) with inactivated WV or intramuscularly (i.m.) with 1 μg of mRNA vaccine encoding SARS-CoV-2 Spike. **(A)** Graphical illustration of the experimental procedure. **(B, C)** Endpoint titers of S protein-specific **(B)** IgA in the nasal wash and **(C)** IgG in the serum were evaluated. The data is presented as the mean ± SD. Each experiment was performed in duplicate. **(B, C)** n = 7–11 per group. **(B, C)** **P < 0.01 as indicated by Tukey’s test. ns, not statistically significant.

Finally, we examined whether systemic priming with an mRNA vaccine, followed by intranasal boosting with the inactivated WV, could enhance antibody responses in the nasal cavity. The mice were divided into four groups (**Figure 5A**). Group 1 was vaccinated intranasally with inactivated WV for priming with no boosting (prime: i.n., boost: none). Group 2 was vaccinated intranasally with the inactivated WV for both priming and boosting (prime: i.n., boost: i.n.). Group 3 was intramuscularly vaccinated with an mRNA vaccine encoding the S protein for priming and intranasally vaccinated with the inactivated WV for boosting (prime: i.m., boost: i.n.). Group 4 was intramuscularly vaccinated with the mRNA vaccine for both priming and boosting (prime: i.m., boost: i.m.). Seven days after final vaccination, the endpoint titers of S-specific IgA in the nasal mucosa of group 2 were found to be significantly higher than those in groups 1 and 4 (**Figure 5B**). Further, the endpoint titers of S-specific IgA in nasal mucosa were significantly higher in group 3 than in groups 1 and 4 (**Figure 5B**). The endpoint titers of S-specific IgG in the serum of group 4 were significantly higher than that of groups 1, 2, and 3 (**Figure 5C**). Further, the endpoint titers of S-specific IgG in the serum of group 3 were significantly higher than those in group 1 but comparable to those in group 2 (**Figure 5C**). Our data suggest that inactivated WV can be used as a booster for individuals already administered the mRNA vaccine.

## Discussion

In this study, we used the Gamma strain (hCoV-19/Japan/TY7-503/2021) of SARS-CoV-2 as the seed virus to generate inactivated WV. Gamma strains were prevalent when this study was initiated at the end of the year 2020 (57), and we used this strain as the seed virus. We demonstrated that virus particles inactivated using β-propiolactone retained the protein composition on the virus surface and the structure of the virus. In previous reports, using TEM analysis, loss of S protein was observed when Wuhan strains were inactivated (58). In contrast, S proteins have been detected on the surface of inactivated WV in another report (10). Differences from existing reports may be attributed to virus strain and the conditions for ultracentrifugation and inactivation, but the full picture is still unclear. Furthermore, we discovered that 3 μg of inactivated WV in the vaccine contained approximately 2.1 μg of S protein. In general, the amount of antigen used for intranasal vaccines is approximately 1-3 μg (33, 37). Therefore, the amount of inactivated WV used in this study appears to be an appropriate dose. In addition, we demonstrated that the levels of nasal IgA and the percentage of GC B cells in the nasal passage were higher after nasal vaccination than subcutaneous vaccination. In contrast, in the neutralization study using nasal wash, the results showed a slight but not significantly different protective effect compared to the control group due to its poor sensitivity. We believe that these results indicated that nasal IgA may contribute to protection against SARS-CoV-2.

B cells within GC undergo robust proliferation and differentiation processes, such as class switch recombination, somatic mutations, and affinity selection, to produce high-affinity protective antibodies (59–62). B cells within the GC in the nasal passage increased upon nasal administration of inactivated WV, which may be involved in enhancing nasal IgA responses. While we did not assess S-specific B cell responses in GC, nasal vaccination of inactivated WV might induce it in the nasal passage; it may be beneficial to evaluate it in the future. In contrast, we investigated the T-cell response induced by nasal or subcutaneous vaccine. IL-13-secreting cells in the spleen in subcutaneous vaccine with alum as a typical T helper 2 (Th2)-inducing adjuvant were higher than that in nasal vaccine with inactivated WV. When regarding the development of vaccines for SARS-CoV-2, it is important to consider the induction of vaccine-associated enhanced respiratory disease (VAERD), such as lung eosinophilic immunopathology, as a result of the Th2-dominant adaptive immune response (33). Our findings revealed that Th1/Th2 ratio in the nasal vaccine with inactivated WV was lower than that in the subcutaneous vaccine, indicating Th2-dominant immune responses in the nasal vaccine, although the level of IL-13 was significantly lower in the nasal vaccination group than in the subcutaneous vaccination group. While the Th1/Th2 ratio is also important for VAERD induction, strong Th2-dominant immune responses are also required for VAERD induction (63). Therefore, we believe that it is highly unlikely that VAERD would be induced by an inactivated WV nasal vaccine because the Th2 immune responses induced by it are very low.

We demonstrated that the protective efficacy of the nasal vaccine against a viral challenge in the upper respiratory tract was superior to that of the subcutaneous vaccine. This suggests that IgA must be induced in the upper respiratory tract by vaccines to effectively protect against SARS-CoV-2 replication in the airways. In general, influenza virus-specific IgA has stronger cross-protective activity than that of IgG (29, 31, 64, 65). Furthermore, in SARS-CoV-2 infection, virus-specific IgA induced by infection and nasal vaccination were protective against several SARS-CoV-2 variants (33, 66). Further, nasal vaccination against SARS-CoV-2 with adenovirus vector-based vaccines has been shown to reduce viral transmission compared to that with parenteral vaccines in several preclinical studies (67–69). Therefore, a nasal vaccine containing inactivated virus may protect against various strains of SARS-CoV-2 infection and transmission. In contrast, the virus titer in the nasal turbinate from the subcutaneous vaccine group was significantly lower than that in the unimmunized control group, but not as much as that in the nasal vaccine group. The subcutaneous vaccine failed to induce S-specific IgA in the nasal, but strongly elicited S-specific IgG in the nasal mucosa, suggesting that S-specific IgG in the nasal mucosa likely contributes partially to prevent upper respiratory tract infection. The neonatal Fc receptor (FcRn) is reportedly involved in the transport of IgG across cellular barriers (70). Possibly, strongly elicited S-specific IgG in the blood following subcutaneous vaccination may be transported into the nasal cavity *via* FcRn.

In a lower respiratory tract challenge with a low titer of SARS-CoV-2 MA10, both nasal and subcutaneous vaccines completely protected against weight loss and death. However, the nasal vaccine showed weight loss during a high-titer SARS-CoV-2 MA10 challenge, whereas the subcutaneous vaccine group showed no weight loss. This may be because the nasal vaccine also induces virus-specific IgG in blood, but not as much as the subcutaneous vaccine. However, many pathogens, including SARS-CoV-2 and the influenza virus, cause upper respiratory tract infections in humans. Therefore, protecting the upper respiratory tract using a nasal vaccine is a useful approach for preventing the progression of symptoms to the lower respiratory tract.

To date, mRNA vaccines against the SARS-CoV-2 Wuhan strain have been used worldwide (71). Many people worldwide have been infected with SARS-CoV-2. This indicates that many people already have immunity against SARS-CoV-2. Therefore, we used an mRNA vaccine encoding SARS-CoV-2 Wuhan strain Spike and evaluated the potential use of an inactivated WV as a nasal booster vaccine. A nasal vaccine with inactivated WV increased S-specific nasal IgA and blood IgG levels in mice previously administered the mRNA vaccine. A recent study showed that intranasal vaccination with the S protein induces robust production of S-specific IgA in the respiratory tract by leveraging the pre-existing immunity generated by the mRNA vaccine (37). These findings suggest that systemic priming, such as vaccination or infection, followed by intranasal boosting with inactivated WV, results in enhanced systemic and mucosal immunity, and that inactivated WV can be useful as a booster vaccine to protect against virus transmission and the severity of infection.

Several challenges need to be addressed in the future. We did not assess respiratory tissue-resident T cell responses in this study. In addition, we did not evaluate immune responses to other antigens, such as membrane and nucleocapsid proteins. DNA vaccines expressing the membrane protein partially protect mice from SARS-CoV-2 (72). Furthermore, nucleocapsid protein is a potential target for the development of a new generation of vaccines (73, 74). The inactivated WV nasal vaccine may also induce immune responses to these antigens.

In conclusion, we demonstrated that a nasal vaccine with inactivated WV can be a potential means of preventing future COVID-19 epidemics. At present, sufficient IgG may be present in blood because of vaccine and virus exposure; therefore, the use of inactivated WV as a booster vaccine may be extremely effective.

## Data availability statement

The raw data supporting the conclusions of this article will be made available by the authors, without undue reservation.

## Ethics statement

The animal study was approved by the ethical treatment of animals and were approved by the Animal Care and Use Committee of the Research Institute for Microbial Diseases, Osaka University, Japan. The study was conducted in accordance with the local legislation and institutional requirements.

## Author contributions

NT, ST, and YY designed the experiments and interpreted the results. NT, ST, MW, JA, HT, and CO performed the experiments and collected and analyzed the data. TO, TH, HA, and YM provided technical support and conceptual advice. ST and YY drafted the manuscript. YY supervised the study. All authors contributed to the article and approved the submitted version.
