# Supplementary figures and images for: A nasal vaccine with inactivated whole-virion elicits protective mucosal immunity against SARS-CoV-2 in mice
Source: Front Immunol. 2023 Aug 31;14:1224634. doi: 10.3389/fimmu.2023.1224634 (PMC10500122; doi:10.3389/fimmu.2023.1224634)

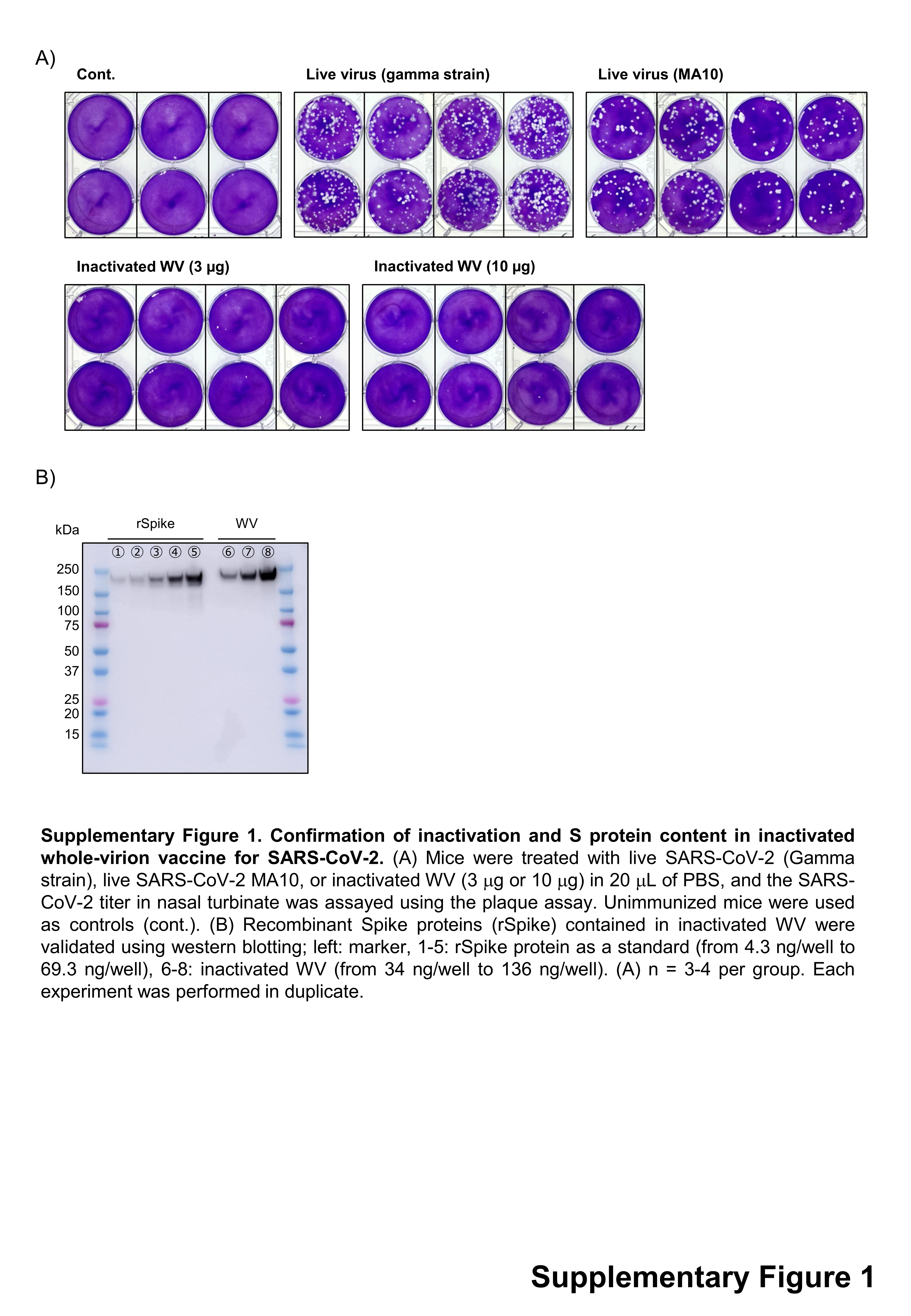

Supplement: Supplementary file 1 [file Image_1.tif]

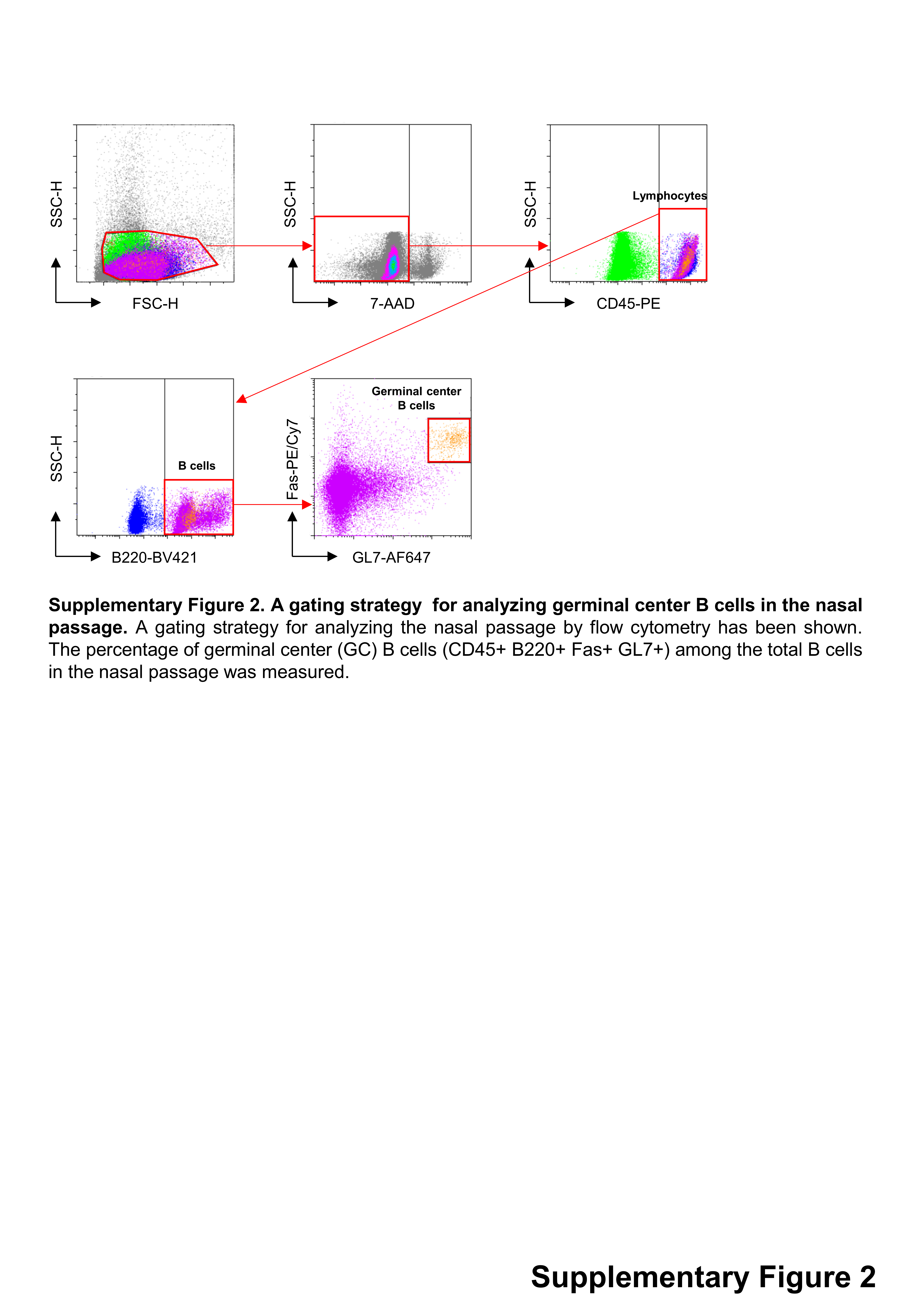

Supplement: Supplementary file 2 [file Image_2.tif]
